# Sex‐based differences in severity and mortality in COVID‐19

**DOI:** 10.1002/rmv.2223

**Published:** 2021-03-01

**Authors:** Mustafa Alwani, Aksam Yassin, Raed M. Al‐Zoubi, Omar M. Aboumarzouk, Joanne Nettleship, Daniel Kelly, Ahmad R. AL‐Qudimat, Ridwan Shabsigh

**Affiliations:** ^1^ Surgical Research Section Hamad Medical Corporation Doha Qatar; ^2^ Jordan University of Science and Technology School of Medicine Irbid Jordan; ^3^ Department of Surgery Hamad Medical Corporation Division of Urology/Andrology & Men's Health Doha Qatar; ^4^ Center of Medicine and Health Sciences Dresden International University Dresden Germany; ^5^ Department of Chemistry Jordan University of Science and Technology Irbid Jordan; ^6^ Department of Surgery Hamad Medical Corporation Doha Qatar; ^7^ College of Medicine Qatar University Doha Qatar; ^8^ College of Medicine University of Glasgow Glasgow UK; ^9^ Department of Oncology and Metabolism Medical School University of Sheffield Sheffield UK; ^10^ Biomolecular Research Centre Sheffield Hallam University Sheffield UK; ^11^ Department of Urology Columbia University College of Physicians and Surgeons New York New York USA

**Keywords:** COVID‐19, dimorphism, sex

## Abstract

The current coronavirus disease (COVID‐19) pandemic caused by novel severe acute respiratory syndrome coronavirus 2 (SARS‐CoV‐2) has a male bias in severity and mortality. This is consistent with previous coronavirus pandemics such as SARS‐CoV and MERS‐CoV, and viral infections in general. Here, we discuss the sex‐disaggregated epidemiological data for COVID‐19 and highlight underlying differences that may explain the sexual dimorphism to help inform risk stratification strategies and therapeutic options.

**Abbreviations**
ACE2: Angiotensin converting enzyme‐2
ARDS: acute respiratory distress syndrome; Acute respiratory distress syndrome
ARDS: acute respiratory distress syndrome; Acute respiratory distress syndrome
Cav1: Caveolin 1
CD: Cluster of Difference
CDC: Centers for Disease Control and Prevention
CFR: case fatality ratio
COCP: combined oral contraceptive pill
COPD: Chronic obstructive pulmonary disease
CXCR3: Chemokine receptor
DCs: Dendritic Cell
DDX3Y: DEAD‐Box Helicase 3 Y‐Linked
GWAS: Gene‐wide association study
ICU: Intensive care unit
IFN α: Interferon α
IFN β: Interferon β
IFN: Interferon
IL: Interleukin
IL‐1β: Interleukin1β
IL4: Interleukin4
IL6: Interleukin6
IL7R: Interleukin‐7 receptor
IL8: Interleukin8
NF‐κB: Nuclear factor kappa B
PAMPs: Pathogen‐associate molecular patterns
pDCs: Plasmacytoid dendritic cell
SARS‐CoV: severe acute respiratory syndrome coronavirus 2
T2DM: Type 2 Diabetes Mellitus
Tfh: T follicular helper
Th1: T helper type 1
Th2: T helper type 2
TLR: Toll‐like receptors
TLR7: Toll‐like receptors7
TMPRSS2: Transmembrane protease serine 2
vWF: von Willebrand factor

## 1 INTRODUCTION

The current coronavirus disease (COVID‐19) pandemic, caused by the novel severe acute respiratory syndrome coronavirus 2 (SARS‐CoV‐2), has overwhelmed healthcare systems around the world bringing significant morbidity and mortality. The World Health Organization has declared it to be a public health emergency of international concern. As of 31 October 2020, there have been >45 million confirmed cases reported worldwide, with deaths exceeding 1.18 million and still rising.^1^ Whilst most reported cases of COVID‐19 experience mild to moderate symptoms including fever, persistent cough, loss of taste and smell, or dyspnoea, about 15% of infected adults develop severe pneumonia requiring oxygen supplementation via invasive mechanical ventilators. Among these, 5% progress to a critical stage with acute respiratory distress syndrome (ARDS), hypoxic respiratory failure and multi‐organ failure, that necessitates mechanical ventilation.^2^, ^3^

**FIGURE 1:**
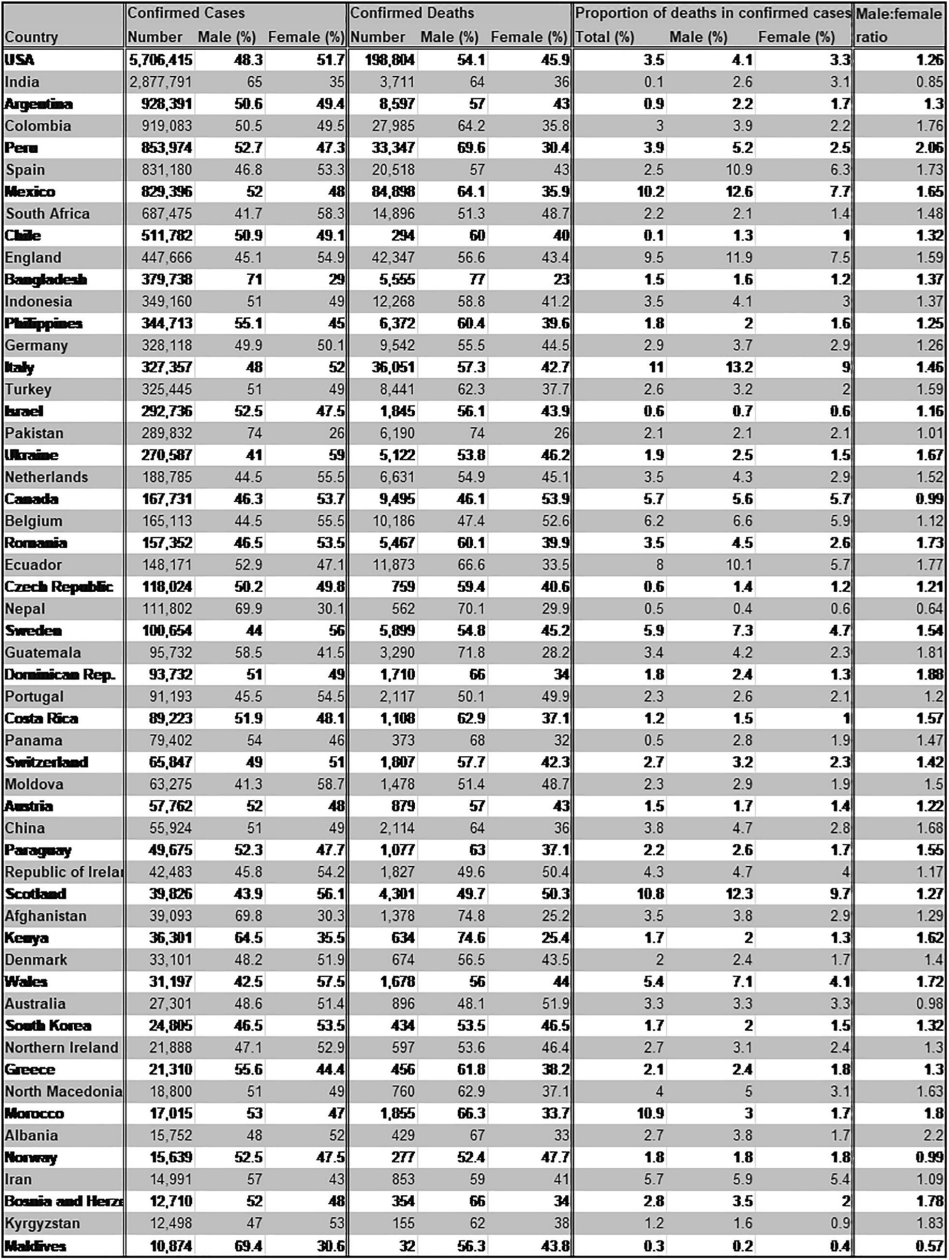
Sex‐disaggregated data of confirmed COVID‐19 cases and deaths from countries with >10,000 cases. Cases and deaths are only reported where sex‐disaggregated data is available, and not total cases. Data from Global Health 50/50 COVID‐19 data tracker as of 31st October 2020.^9^

**FIGURE 2:**
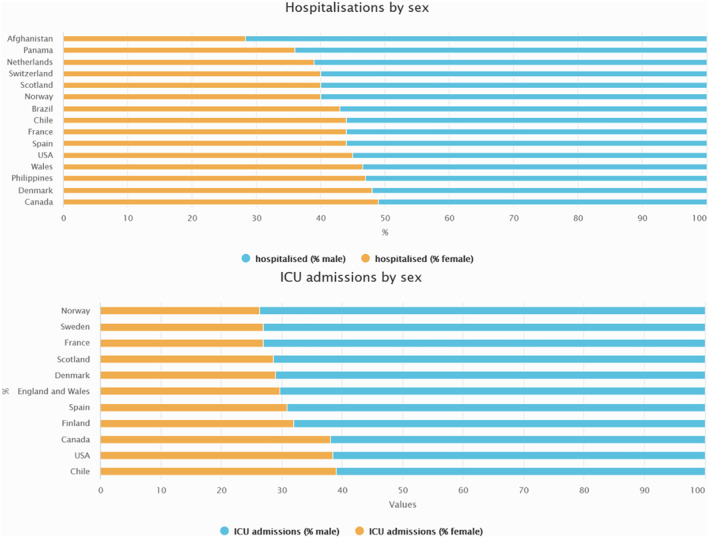
Disease severity is higher in males as measured by hospitalizations and ICU admissions. Graphs reproduced from Global Health 50/50 COVID‐19 data tracker.^9^

Epidemiological data from previous coronavirus epidemics‐SARS‐CoV (2002) and Middle Eastern respiratory syndrome coronavirus (MERS, 2012) highlighted differences in their manifestation based on sex, with men being consistently more severely affected than women.^4^, ^5^, ^6^, ^7^ Early reports of COVID‐19 also suggest a sex imbalance, with men at a higher risk of more severe disease and increased case fatality ratio (CFR).^3^, ^8^ Publicly available sex‐disaggregated data from several governments compiled by the Global Health 50/50 research initiative also show, despite similar numbers of COVID‐19 cases in men and women, an increased fatality rate in men as outlined in Figure 1, with the male:female ratio of deaths among confirmed cases ranging from 1.0 in Pakistan and Canada to 2.1 in Wales.^9^ In addition to fatality, hospitalizations and admissions to intensive care units (ICU) can serve as a measure of disease severity. A review of epidemiological data by Gebhard et al. (2020) comprising confirmed COVID‐19 cases in several countries including China, Italy and Spain show that there were 50% more men requiring hospitalization compared to women, with ICU admission being three to fourfold higher.^10^, ^11^ A meta‐analysis of 15 independent studies that recorded sex disaggregated patient outcomes found men had an odds ratio of 1.31 to develop a severe COVID‐19 infection compared to women.^12^ United States of America has the highest number of COVID‐19 cases to date and early reports by the Centers for Disease Control and Prevention (CDC) across 14 states also observed higher hospitalization rates for men.^13^ A recent count by Global Health 50/50 also confirms this indication of sex imbalance in disease severity across several countries (Figure 2).^9^ There are, however, some limitations to this data set particularly as the interaction between age and sex remains unexplored.

**FIGURE 3:**
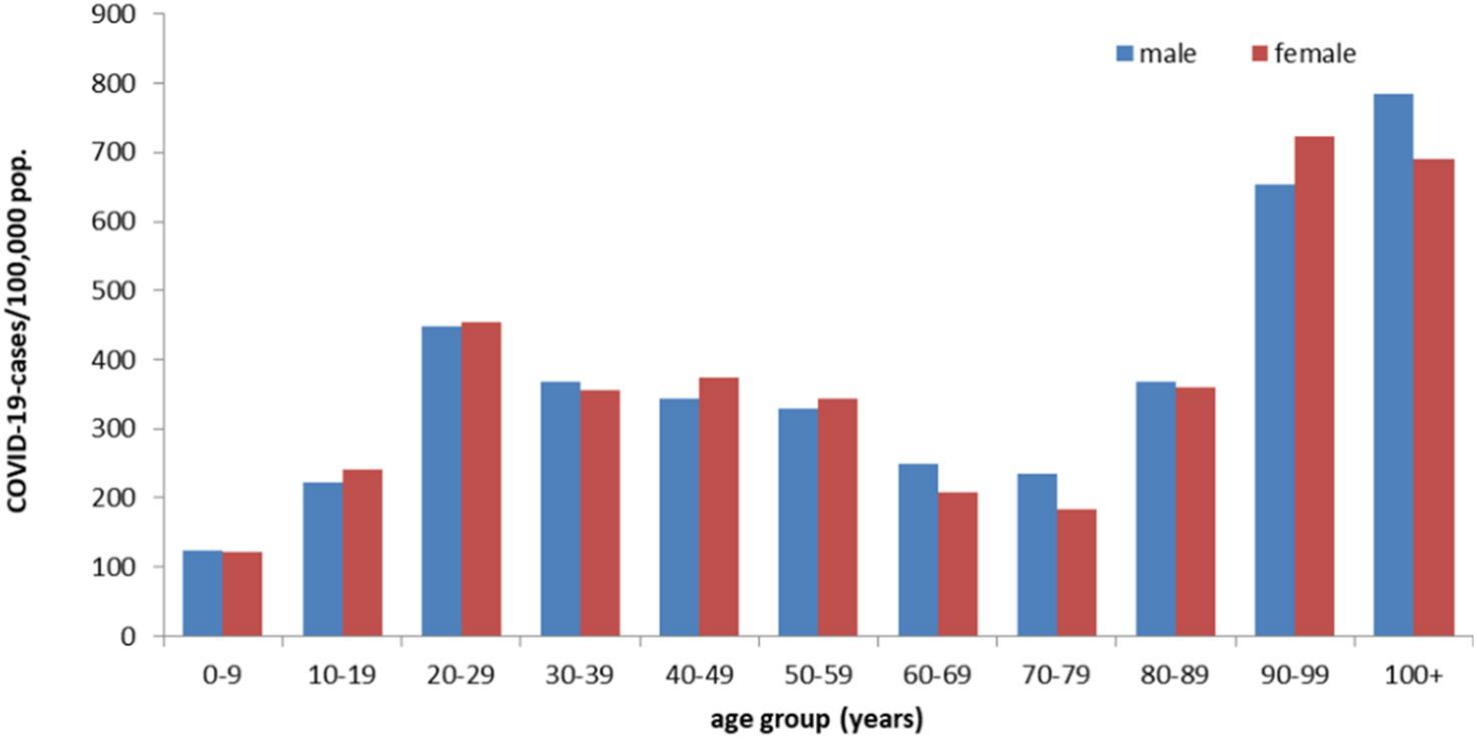
Reported COVID‐19 cases in Germany by age group and gender (*n* = 254,549) (Data accessed on10/09/2020, from https://www.rki.de/EN/Content/infections/epidemiology/outbreaks/COVID‐19/Situationsberichte_Tab.html)

On investigating the magnitude of differences in survival for both sexes in Europe across different age groups, Ahrenfeldt et al. (2020) reported that the relative risk of dying from COVID‐19 is consistently elevated in men across all age groups with the differences increasing until the age range 60–69 years.^14^ Thereafter, the sex difference in survival decreases and was at its lowest for ages 80+.^14^ A study comprising of 227,000 confirmed cases of COVID‐19 pooled from Italy, Germany, Spain and Switzerland also suggested that the sex difference in fatality is most pronounced in the ages 50–59, and decreases subsequently with increasing age.^10^ Interestingly, as per the Global Health 50/50 data, numbers of confirmed cases are similar for men and women, suggesting equal infection rates.^9^ More detailed reports on incidence rates from Switzerland and Germany also suggest similar proportions of COVID‐19 cases in men and women at all age groups (Figure 3), thereby highlighting the worsened prognosis in infected males compared to females.^15^, ^16^

To some extent, sex difference in COVID‐19 expands to affect the male reproductive system either directly or indirectly as some articles suggest.^17^

As vaccines and other treatment modalities are researched and developed in attempt to contain the recurrent surges in infections for this pandemic, better understanding of the sex imbalance and its underlying biology can help inform public health strategies for testing and intervention by stratifying groups at high risk for severe disease; and help improve therapeutic options by allowing gender‐specific targeted treatments. Herein, we discuss several factors that may contribute to the sex differences observed in COVID‐19 patients, including possible biological reasons, contributions of comorbidities, and highlight any role that gender may play.

## 2 SEX DIFFERENCES IN VIRAL ENTRY

Angiotensin converting enzyme‐2 (ACE2) catalyses the conversion of angiotensin‐II to angiotensin (1–7), and plays a vital role in homeostasis of blood pressure, inflammatory responses and blood coagulation.^18^ ACE2 is expressed in a range of tissues including nasal and respiratory epithelial cells, blood vessels and kidneys.^19^, ^20^, ^21^, ^22^ As with SARS‐CoV, membrane‐bound ACE2 serves as a receptor for the SARS‐CoV‐2 spike (S) glycoprotein, facilitating its attachment to the cell surface and subsequent entry.^23^

The expression levels of ACE2 correlate with the risk of COVID‐19 severity, with children who have lower ACE2 expression in their nasal epithelium having a lower risk compared to adults.^24^ However, evidence regarding a sex disparity in ACE2 expression is unclear. A phenomenon, termed X chromosome inactivation, is an epigenetic process that silences one of the two X‐chromosomes in females to maintain balance in gene expression dosage.^25^ The ACE2 gene is located on the X chromosome, and is thought to have higher levels of expression in females. This is because it has been reported that ACE2, frequently ‘escapes’ inactivation that occurs to balance expression dosage between the sexes, has an uncharacteristic heterogeneous pattern of male‐female expression based on the tissues.^25^ In pre‐clinical studies, ACE2 expression has been reported to be higher in female rat lungs and kidneys.^26^, ^27^ Conversely, oestrogen downregulates the expression of ACE2 in vitro and in gonadectomised female mice.^28^, ^29^ In human tissues, studies have suggested no significant difference in the expression of ACE2 for both sexes in respiratory tissues,^30^, ^31^, ^32^ and in circulation are more elevated for male patients with comorbidities such as cardiovascular diseases.^33^ Taken together, the levels of ACE2 expression varies based on tissues and underlying comorbidities and therefore it may not be a strong predictor for disease severity isolation.

Transmembrane protease serine 2 (TMPRSS2) is also vital for viral entry, following its binding to ACE2, by priming the viral S protein (by proteolytic cleavage) and mediating fusion of viral and cell membranes.^34^, ^35^ Indeed, in an in vitro model*,* inhibiting TMPRSS2 activity partially inhibited the entry of SARS‐CoV‐2 into lung epithelial cells.^34^ In vivo, TMPRSS2 deficient mice demonstrated reduced weight loss and inflammatory response in the lungs following SARS‐CoV and MERS‐CoV infections, suggesting decreased severity.^36^

Interestingly, a study by Asselta et al. (2020), which compared the expression of TMPRSS2 in the two sexes from a large Italian cohort observed a higher expression of TMPRSS2 in bronchial epithelial cells in the males compared to females.^31^ TMPRSS2 plays a pivotal role in the development and progression of prostate cancer via gene fusion, and is strongly upregulated in response to androgens.^37^, ^38^ These data suggests that TMPRSS2 expression might mediate the sex disparity observed in severity of COVID‐19. However, it is unclear if androgen signalling can modulate TMPRSS2 expression in respiratory tissues, and whether low level of androgens present in women can maintain TMPRSS2 expression in respiratory tissues. Further research is therefore required to determine if there is a sex‐biased expression and/or regulation of ACE2 and TMPRSS2 that confers increased severity COVID‐19 in males compared to females. This is especially topical with current treatment strategies targeting these proteins.^39^

## 3 SEX DIFFERENCES IN IMMUNE RESPONSES

Sex‐based differences in immune responses have been reported for adults and children,^40^ suggesting an influence of both the sex chromosome and hormones on the immune system. The X chromosome encodes several genes that regulate immune function and is fundamental in shaping sex‐specific immune responses.^41^ As mentioned previously, X chromosome inactivation silences one of the two X‐chromosomes in females to maintain balance in gene expression dosage. This process leads to 50% cells in females having the maternal X chromosome inactivated, whilst the paternal X chromosome is inactivated in the rest, a phenomenon termed as ‘cell mosaicism’.^42^ This provides females with a greater plasticity and adaptability in response to infections, especially in case of X‐linked mutations by expressing the corresponding wild‐type allele on the other X chromosome.^43^ Furthermore, some immune related genes are partially reactivated in female lymphocytes to confer enhanced immunity to infectious diseases.^44^

### 3.1 Role of innate immunity

The sexual dimorphism in immune responses to vaccines and viral infections has been well documented. There is compelling evidence showing that females differ in their innate recognition and response to viral infections, and mount a greater inflammatory and humoral immune response.^45^, ^46^ As a result, both the prevalence and intensity of viral infections are often lower for females.^47^, ^48^, ^49^ Toll‐like receptor are a class of innate immune pattern recognition receptors that recognize bacterial or viral pathogen‐associated molecular patterns. TLR7 is an endosomal receptor expressed constitutively in plasmacytoid dendritic cells (pDCs) and B‐cells,^50^ and is capable of detecting single‐stranded ribonucleic acids from viruses, including coronavirus.^51^ Upon recognition of viral infection, TLR7 triggers an antiviral type I interferon (IFN) response which serves to control viral replication and activate an adaptive immune response to clear the infection.^52^, ^53^

TLR7 is encoded on the X‐chromosome, and is one of the 23% of the X‐linked genes that exhibit incomplete inactivation resulting in increased dosage in females.^54^ Using single‐cell analyses, Souris et al. (2018) demonstrated that TLR7 is transcribed on both X chromosomes in pDCs and B‐cells, and correlates with higher TLR7 protein expression in female leucocyte populations.^55^ This disparity in TLR7 expression enhances innate immune responses to viruses,^56^ and may confer females an advantage as observed with COVID‐19. Indeed, some early COVID‐19 case‐reports suggest a link between loss‐of‐function TLR7 mutations and increased disease severity in young patients.^57^

Interferon α and β (IFNα and IFNβ) are the primary effector cytokines of the type I IFN response downstream of TLR activation, and critical players of the immune system, linking innate to adaptive immunity.^58^ IFNα/β production by pDCs is primarily mediated through the stimulation of TLR7 during viral infection, and is essential for the maturation of DCs to effective antigen‐presenting cells with increased ability to activate T cells.^59^, ^60^, ^61^ Several studies have shown that pDCs from females produce more IFNα/β than males, following TLR7 activation by viral RNA.^56^, ^62^, ^63^ In a study cohort of 50 COVID‐19 patients with mild/moderate to critical severity, Hadjadj et al. (2020) employed an integral approach by conducting in‐depth phenotypical analysis of immune cells, whole‐blood transcriptomics and cytokine measurements.^64^ They observed that the severity of the disease characterized by persistent viral load in the blood and exacerbated inflammation associated with highly impaired type I IFN response, with very low levels of IFNα and no IFNβ.^64^ Taken together, this suggests that females may have a better prognosis following SARS‐CoV‐2 infection partly because of their heightened type I IFN response, and enhancing type I IFN response could serve as a therapeutic possibility for COVID‐19.

Genetically, a British gene‐wide association study attained in critically ill COVID‐19 patients, revealed loss‐of‐function mutation in the one of the responsible genes for IFNα and IFNβ expression called IFNAR2, explaining the lower interferons level in the critically ill patients specifically.^65^

Whilst the initial immune response against the pathogen is vital to protect the host, an overactivation of the response that results overproduction in pro‐inflammatory cytokines causes immunopathology leading to multiple organ failure and ultimately death. In a recent study, Liu et al. (2020) evaluated the severest multi‐organ dysfunctions during entire hospitalization between males and females which helps predict in‐hospital death.^66^ They observed that whilst multiple key factors that characterize a ‘cytokine storm’ were elevated (pro‐inflammatory cytokines such as TNFα, IL6 and IL8) in females during the acute infection phase, these mediators were lower when measured over the whole duration of hospitalization, possibly offering a survival advantage. However, when the infection persisted, the heightened immune response in females led to more ferocious organ injuries and their survival advantage diminished.^66^

### 3.2 Role of adaptive immunity

In general, females mount a much stronger cellular and humoral immune response post viral infections,^67^, ^68^, ^69^ and vaccinations.^70^ T cells play an integral role in cell mediated immune response, with CD4+ T cells orchestrating the B cell response for antibody production and CD8+ T cells responsible for the killing of infected cells and reducing viral burden. Akin to SARS‐CoV,^71^ several reports following SARS‐CoV‐2 infection describe a correlation of COVID‐19 severity with lymphopenia, and drastically reduced circulating CD4+ and CD8+ T cells.^72^, ^73^, ^74^ In a longitudinal study comparing immune responses between sexes, Takahashi et al. (2020) observed that whilst T cell lymphopenia occurred in both sexes, female patients mounted a more robust T cell activation, particularly for CD8+ T cells.^75^ A balance between the CD4+ pro‐inflammatory T helper type 1 (Th1) and anti‐inflammatory type 2 (Th2) subtypes is vital for regulating the immune response and resolving of the infection without damaging host tissue. Studies with SARS‐CoV and MERS‐CoV describe upregulation of Th1 cytokines, dysregulating the Th1/Th2 balance.^76^, ^77^ Sexual dimorphism in Th1 and Th2 responses based on the stages of infection have been reported previously,^78^ and further research is needed to elucidate if it plays a role in COVID‐19.

T follicular helper (Tfh) cells, are a subset of CD4+ T cells, responsible for the differentiation of B cells into plasma cells and memory B cells. In patients with severe disease particularly, the improved outcomes for females can also be attributed to higher levels of circulating IgG antibodies against SARS‐CoV‐2 as observed by Zeng et al. (2020).^79^ Furthermore, in a comprehensive analysis of sex differences in B‐cell gene expression, Fan et al. (2014) found over 350 differentially expressed genes between males and females.^80^ These include upregulation of immune response genes such as Cav1, CXCR3, and downregulation of inflammatory genes such as IL7R and DDX3Y, and may account for some of the observed sex bias observed in COVID‐19.^80^ Taken together, the reports suggest that the immune landscape in COVID‐19 is considerably different between the two sexes and may contribute to the higher susceptibility observed in males. These, along with genes such as ACE2 and TLR7 that escape inactivation, cause a gene dosage imbalance between the sexes and may contribute to the immune disparity.

### 3.3 Role of sex hormones

The role of sex hormones in regulating immunity is well characterized, and is likely to play a role in differences in the severity of COVID‐19 between males and females. Oestrogen has a dual effect based on its levels. At low doses similar to those in post‐menopausal women, oestrogen is immuno‐stimulatory and induces differentiation of inflammatory dendritic cells, higher production of IL‐4 and IFNα, and an increased Th1 type and cell mediated responses. Conversely, at higher doses observed in premenopausal women, oestrogen promotes anti‐inflammatory Th2 responses and is inhibitory to the pro‐inflammatory innate immune response.^78^, ^81^, ^82^, ^83^ Indeed, Channappanavar et al. (2017) demonstrated that mortality in female mice infected with SARS‐CoV increased following ovariectomy or exposure to oestrogen receptor antagonist suggesting a protective role of oestrogen receptor signalling.^84^ A similar protective effect of oestrogen has also been suggested in a recent pre‐print study which investigated the association of oestrogen with severity of COVID‐19 symptoms.^85^ The study observed a higher risk in post‐menopausal women; and in younger women who did not take the combined oral contraceptive pill (COCP) compared to those of similar aged women taking COCP.^85^ A similar observation was reported by Ding et al. (2020), who showed that post‐menopausal women were at a greater risk of hospitalization, and that oestrogen levels had a protective effect against disease severity.^86^ This protective effect of oestrogen was attributed to reduced levels of inflammatory IL‐6, IL‐8 and TNFα.^86^

Early reports from China, Germany and Italy have suggested that low testosterone levels strongly correlate with disease severity and the need for intensive care in male patients.^87^, ^88^ Testosterone immunosuppresses,^89^ by reducing the production of pro‐inflammatory IL‐6, IL‐1β and TNFα via inhibition of the NF‐κB pathway.^90^ In fact, IL‐6 is a key mediator of disease progression to ARDS in COVID‐19, and a clinical trial of tocilizumab, an IL‐6 receptor blocker, is approved in China for patients with severe disease.^91^ This suggests that the role of testosterone on the immune response to SARS‐CoV‐2 may be beneficial to patient outcomes potentially suppressing uncontrolled inflammatory responses. Conversely, men with higher levels of testosterone have weakened immunity and produce the lowest antibody responses to annual flu vaccinations.^92^

Low serum testosterone can increase the expression of ACE‐2 receptors and TMPRSS2, with patients reportedly developing severe manifestations of COVID‐19 infections which require assisted ventilation as a result of the upregulation of ACE‐2 receptors in lower respiratory cells increasing risk of lung damage and respiratory muscle catabolism.^93^, ^94^ Reduced testosterone in men can also inhibit pulmonary endothelial cell function as SARS‐CoV‐2 reduces ACE‐2 concentrations by binding and increasing angiotensin‐II while lowering angiotensin 1–7. As a result of this process superoxide species become increased, leading to oxidative stress induced endothelial cell dysfunction and localised inflammation.^95^, ^96^ Consequently, von Willebrand factor expression can increase and development of thrombosis in the alveolar capillaries, a precursor of ARDS, ensues.^97^, ^98^, ^99^

Testosterone levels are known to decline following onset of disease, and in particular, during infections with low testosterone often considered a marker of ill health.^100^ This may have evolutionary origins to move energy away from high‐energy consuming anabolic processes to allow instead most energy for strengthening the immune response. It has been suggested that COVID‐19 might deteriorate serum testosterone level in SARS‐CoV‐2 infected male patients and that a lower pre‐infection testosterone may significantly increase the risk of ICU transfer and mortality.^101^ The authors additionally propose possible improvement in clinical outcomes with the testosterone treatment in SARS‐CoV‐2 infected hypogonadal male patients. However, due to the varying effects on different aspects of the immune system (it is not likely that testosterone's anti‐inflammatory effects would reduce all parts of immune function equally) it would be necessary to look at testosterone effects on various functions of both innate and adaptive immunity in a variety of contexts to elucidate its therapeutic potential, as well as testosterone deficiency in hypogonadism contribute to increase the risk of comorbidities such as type 2 diabetes mellitus (T2DM), and cardiovascular diseases.^101^, ^102^, ^103^ Thus, increase the rate of ICU admissions and mortality in COVID‐19 patients as Cayan S et al. suggests.^101^

Furthermore, a systemic review spotlights in the acute management of COVID‐19, T2DM and hypogonadism suggests that treating testosterone deficient COVID‐19 patients with testosterone might be considered in the future as Testosterone reduces the risk of T2DM, and boosts the body inflammatory response against the virus acutely.^102^ However, further studies required in order to reveal the optimal effect of Testosterone replacement therapy.

## 4 SEX AND GENDER RELATED RISK FACTORS

Clinical data has highlighted that specific comorbidities increase the risk of COVID‐19 severity. Guan et al. (2020) showed that COVID‐19 patients with comorbidities have a poorer prognosis, and that greater number of comorbidities correlate to poorer clinical outcomes.^2^ Specific comorbidities associated with poorer patient outcomes included chronic obstructive pulmonary disease (COPD), Obesity, diabetes, cerebrovascular disease, cancer and hypertension.^2^, ^102^, ^103^, ^104^ Globally, men have more of these morbidities than women,^105^ placing them at a higher risk for severe disease.

In addition to biological sex‐based differences, gender as defined as the social and cultural norms, roles, attributes and behaviours that society considers appropriate for males and females, is likely to play a role in the incidence and fatality of COVID‐19. A meta‐analysis by Zhao et al. (2020) which analysed data from 1726 patients showed that smoking has a significant association with COVID‐19 severity, with odds ratio of 2.0 (95%CI 1.3‐3.1).^106^ This observation has since been confirmed by several studies that also reported increased disease severity and death in COVID‐19 patients who smoke, potentially related to higher expression of ACE2.^107^, ^108^ Indeed, gender differences in smoking rate between men and women has been suggested to contribute to their predisposition to COVID‐19 progression.^109^ However, a male bias is often still observed in countries reporting equal rates of current smokers between the genders, and large variations across age and ethnicity confound this relationship resulting in review of the current literature not supporting smoking as a predisposing factor in men for COVID‐19 incidence or severity.^109^ Other gender‐based differences include delay in accessing health services by men that lead to higher fatality, as suggested from the data of the Ebola outbreak.^110^ Furthermore, handwashing behaviour, which is the primary public health message in this pandemic, also exhibits a sex difference with women being more frequent adherers to guidelines.^111^ Therefore, understanding the sex differences in COVID‐19 severity and mortality requires recognition of both the biological and the social factors that may play a role.

### 4.1 Sex disparity in ‘long–COVID’

It is becoming evident that the impact of the SARS‐CoV‐2 pandemic is likely to be much larger due to the long term persistence of symptoms in patients following the initial acute stage. In a subset of COVID‐19 patients, a syndromic state post the acute symptomatic phase has been reported which includes a wide range of symptoms such as dyspnoea, extreme fatigue, tachycardia and mental fog.^112^, ^113^, ^114^ This prolonged symptomatic phase (beyond 3‐weeks) is being referred to as ‘Long‐COVID’, ‘Long‐haulers’ or ‘Chronic COVID Syndrome’, and is still poorly understood.

A recent report describing data collected from over 4000 patients using a mobile application showed that symptoms persist for 28 days in 13% patients, of which 4.5% and 2.3% experienced symptoms for 56 and 94 days respectively.^115^ The study also reported that age significantly associated with long‐COVID, rising from 10% in 18–49 year olds, to 22% in those above 70. Interestingly, and conversely to the acute phase, long‐COVID seems to affect women disproportionately (14.9%) compared to men (9.5%), with females aged 50–60 having the highest odds ratio, although this sex effect was not significant in the older age‐group (>70).^115^ Whilst the aetiology of the syndrome and reasons for any sex disparity needs further research, a pre‐existing asthma condition, which is more common in women, increased the odds of having long‐COVID.^115^, ^116^

## 5 CONCLUSION

The sex disparities in COVID‐19 severity and mortality are multifactorial, may also be resulted from the sex‐difference comorbidities and behaviours, thus underline the need to collect sex and age‐disaggregated data to better understand disease pathology and guide clinical care. It has also highlights the need to incorporate sex and gender analyses in any therapeutic strategies under consideration, and vaccine development protocols. Furthermore, the consistencies with previous coronavirus pandemics suggest that the public health policies and risk stratification should take sex into consideration for any future outbreaks.

## AUTHORS CONTRIBUTIONS

Ridwan Shabsigh: Supervision, Concept, Literature Search, Manuscript Writing, Manuscript Grammatically Revision, Critical Revision. Aksam Yassin: Supervision, Concept, Manuscript Writing, Manuscript Grammatically Revision, Critical Revision. Mustafa Alwani: Concept, Literature Search, Manuscript Writing, Manuscript Grammatically Revision, Final Revision (Editor and Reviewer Comments). Joanne Nettleship: Literature Search, Daniel Kelly: Literature Search, Manuscript Grammatically Revision. Raed M. Al‐Zoubi: Manuscript Writing. Omar M. Aboumarzouk: Final Revision (Editor and Reviewer Comments). Ahmad R. AL‐Qudimat: Final Revision (Editor and Reviewer Comments).

## Data Availability

The data that support the findings of this study are openly available in [Global Health 50/50] at [globalhealth5050. org], reference number.^9^
